# ﻿Chromatin diminution as a tool to study some biological problems

**DOI:** 10.3897/compcytogen.17.112152

**Published:** 2024-02-08

**Authors:** Andrey Grishanin

**Affiliations:** 1 Papanin Institute for Biology of Inland Waters, Russian Academy of Sciences, 152742 Borok, Yaroslavl Prov., Russia Russian Academy of Sciences Borok Russia; 2 Department of Biophisics, Faculty of Natural and Engineering Sciences, Dubna State University, Universitetskaya 19, 141980, Dubna, Moscow Prov., Russia Dubna State University Dubna Russia

**Keywords:** *C*-value enigma, differentiation, evolution, genome reorganization, recombination, speciation

## Abstract

This work reveals the opportunities to obtain additional information about some biological problems through studying species that possess chromatin diminution. A brief review of the hypothesized biological significance of chromatin diminution is discussed. This article analyzes the biological role of chromatin diminution as it relates to the *C*-value enigma. It is proposed to consider chromatin diminution as a universal mechanism of genome reduction, reducing the frequency of recombination events in the genome, which leads to specialization and adaptation of the species to more narrow environmental conditions. A hypothesis suggesting the role of non-coding DNA in homologous recombination in eukaryotes is proposed. *Cyclopskolensis* Lilljeborg, 1901 (Copepoda, Crustacea) is proposed as a model species for studying the mechanisms of transformation of the chromosomes and interphase nuclei structure of somatic line cells due to chromatin diminution. Chromatin diminution in copepods is considered as a stage of irreversible differentiation of embryonic cells during ontogenesis. The process of speciation in cyclopoids with chromatin diminution is considered.

## ﻿Introduction

All organisms exhibit genome variability produced by mutations, recombinations, deletions, insertions, mobile elements, etc. A small number of animals additionally exhibit a special, hard-coded form of genome modification called chromatin diminution, DNA elimination, programmed DNA elimination (PDE). As a result of this complex, genetically determined process chromosomes, and fragments of chromosomes undergo elimination. The phenomenon of chromatin diminution has a long history and was discovered by Theodor Boveri (1887). Suffice it to mention that only a year after the discovery of chromatin diminution, Waldeyer (1888) introduced the term chromosome into scientific use. Interest in chromatin diminution revitalized in the second half of the 20^th^ and early 21^st^ centuries (Beermann 1959, 1977, 1984; White 1959; Stich 1962; Painter 1966; Kunz et al. 1970; Geyer-Duszynska 1959, 1961; Bantock 1970; Kloetzel 1970; Ammermann 1971; Ammermann et al. 1974; Wyngaard and Chinnappa 1982; Tobler et al. 1985; Tobler 1986; Bennet 1987; Etter et al. 1991; Prescott 1992; Grishanin et al. 1996; Dorward and Wyngaard 1997; Wyngaard and Rasch 2000; Kloc and Zagrodzinska 2001; Rasch and Wyngaard 2006; Wang and Davis 2014; Zagoskin and Wang 2021). Studies of chromatin diminution (elimination) attracted the attention of an increasingly wide range of scientists, and information about this amazing phenomenon began to quickly accumulate. Review articles devoted to chromatin diminution (elimination) shows how comprehensive research on this phenomenon has become (Raikov 1976; Ammermann 1985; Prescott 1992; Tobler et al. 1992; Goday and Pimpinelli 1993; Grishanin et al. 2006b; Grishanin 2014; Wang and Davis 2014; Dedukh and Krasikova 2021; Drotos et al. 2022; Kloc et al. 2022).

Chromatin diminution in metazoans is the removal of chromosomal material (mostly heterochromatin) from the cells of the somatic line in early embryogenesis; chromatin diminution (programmed DNA elimination) in Protozoa is the removal of entire chromosomes or of sequences interspersed among genic loci in the somatic nucleus. The process of chromatin diminution is species-specific. The diploid number of chromosomes after diminution processes can remain the same or change. The biological role of chromatin diminution remains unknown. Despite being studied for over 100 years, chromatin diminution, in the author’s opinion, is an example of one of the most underestimated biological phenomena. The purpose of this work is to show that chromatin diminution is not only interesting as a biological phenomenon, but also provides researchers with a unique opportunity to work with species in which genome size changes during ontogeny, in some cases by more than 90%, which provides additional advantages when studying various biological structures and processes.

## ﻿Biological roles of chromatin diminution

The biological role of chromatin diminution remains open. Theodor Boveri was the first to suggest a biological role for the eliminated chromatin. He suggested that the eliminated chromatin has important functions for germline cells, since centrifugation of *Parascarisequorum* Goeze, 1782 (Ascaridida, Nematoda) embryos in the early stages of development initiates diminution in all cells of the embryo, including germline cells (Boveri 1887). Therefore, according to the Boveri hypothesis, chromatin diminution is necessary to determine the direction of development. Evidence that chromatin diminution in parasitic nematodes involves the loss of unique genes from the germline cells and represents the first molecular evidence for Boveri’s hypothesis (Etter et al. 1991, 1994; Spicher et al. 1994; Huang et al. 1996). Sigrid Beermann (1977) suggested, considering the chromatin diminution process either as an extreme case of chromatin inactivation, or as a rare variant of chromosomal polymorphism, which leads to the development of heterochromatic blocks in some species of Cyclopoida (Copepoda, Crustacea). One function of DNA eliminated during chromatin diminution may be to control transcription in germline cells, to regulate meiosis, and to regulate replication and transcription processes (Ammermann 1985). Goday and Pimpinelli (1993) consider chromatin diminution as a mechanism for regulating quantitative changes in gene products during ontogenesis. Others scientists suggest that the role of the eliminated DNA is the regulation of recombination processes and the formation of bivalents during meiosis (Müller and Tobler 2000; Staiber and Wahl 2002). The retention of satellite DNA in the germline of *Ascaris* Linnaeus, 1758 may contribute to meiotic homologous recombination, genome evolution, or serve as chromatin spacers, scaffolds, or impact 3D genome organization (Shatskikh et al. 2020). The detection in the eliminated fraction of the *Ascarislumbricoides* Linnaeus, 1758 (Ascaridida, Nematoda) genome of the gene encoding the ALEP-1 ribosomal protein supports the idea that chromatin diminution is an alternative way of regulating gene activity (Tobler et al. 1992). Others hypothesize that nematodes use chromatin diminution to silence germline-expressed genes in the soma and for sex determination for some species of Strongylidae (Rhabditida, Chromadorea) (Albertson et al. 1979; Streit et al. 2016). Standiford (1988), researching oogenesis in *Acanthocyclopsvernalis* Fischer, 1853 (Crustacea, Copepoda) suggested the hypothesis that rDNA sequences are lost during chromatin diminution. The subsequent research of chromatin diminution reported gene deletion during this phenomenon in many taxa (Zagoskin and Wang 2021). For example, ribosomal RNA (rRNA) genes are eliminated in *Cyclopskolensis* Lilljeborg, 1901 (Copepoda, Crustacea) (Zagoskin et al. 2010). It is assumed that a large number of copies of rRNA genes is required only in gametogenesis and in the early stages of development. For later developmental stages, a large number of ribosomal DNA copies may not be necessary. It is also possible that chromatin diminution removes only inactive copies of rDNA. Moreover, the number of rDNA copies can be adjusted according to the genome size using chromatin diminution, since the number of rDNA copies positively correlates with the size of the eukaryotic genome (Prokopowich et al. 2003). The hypothesis of Goday and Pimpinelli (1993) connects the elimination of chromatin in presomatic cells of nematodes with an increase in the ploidy of individual somatic cells of the adult organism and considers chromatin diminution as a mechanism for regulating gene expression by regulating chromatin amount or gene dosage during ontogeny. The eliminated DNA of the parasitic nematode *Ascaris* contains genes (1000 genes in total) that are predominantly expressed in the germline (Wang et al. 2020). However, considering that genes make up only a small part of the eliminated sequences, it can be concluded with high probability that the removal of genes is not the main goal of chromatin diminution (Zagoskin and Wang 2021).

As a result of chromatin diminution in *Ascaris* chromosomes, both preserved and eliminated chromosomes acquire new telomeres (Wang et al. 2020). It is also proposed that a decrease in genome size due to chromatin diminution leads to a decrease in cell size and a shortening of the cell cycle, which in turn causes a decrease in body size and the achievement of sexual maturity at an earlier age (Gregory and Hebert 1999; Gregory 2001; Wyngaard et al. 2005). A hypothesis stating that the elimination process ensures the maintenance of a functional somatic genome and concomitantly allows extremely rapid and profound changes in the germ line genome is presented, thereby allowing the development of new germ line specific functions and providing a selective advantage for the chromatin diminution in nematodes during subsequent evolution (Bachmann-Waldmann et al. 2004).

In some species, representatives of the order Diptera (Cecidomyiidae, Sciaridae, Chironomidae), elimination of individual chromosomes or entire chromosome sets is observed in the process of sexual differentiation; elimination of chromosomes is preceded by their heterochromatinization (Geyer-Duszynska 1959, 1961; White 1959; Hartl and Brown 1970; Fux 1974; Matuszewski 1982; Jazdowska-Zagrodzinska et al. 1992).

The most complete list of existing hypotheses about the biological significance of programmed DNA elimination (chromatin diminution) suggests the following functions: gene silencing and regulation, nucleotypic effects, mutation rate reduction, and energetic benefits (Grishanin 2014; Wang and Davis 2014; Dedukh and Krasikova 2021; Drotos et al. 2022; Kloc et al. 2022).

## ﻿Causes and consequences of changes in the structure of interphase nuclei during chromatin diminution in *Cyclops* Müller, 1785 (Copepoda, Crustacea)

The study of chromatin diminution in *Cyclopsstrenuusstrenuus* Fisher, 1851 showed that throughout the prediminution interphase, the nucleus of somatic cells has a weak uniform color. Only 20 minutes before the start of division, numerous lumps of condensed chromatin appear in the nucleus, distributed along the periphery of the nucleus (Beermann 1977). A similar pattern was observed in *C.kolensis* (Grishanin 1995). The nuclei of embryonic cells of *C.kolensis* in the early interphase of the first cleavage divisions have a relatively weak homogeneous color; heterochromatinized structures and chromocenters are absent, which is manifested on preparations stained both by the Feulgen method and studied using the electron microscope. After chromatin diminution, chromatin remains scattered throughout the nucleus, but is interspersed with more condensed heterochromatic segments. The chromocenters become detectable, and part of the chromocenters adjoin the nuclear membrane (Grishanin 1995). A similar picture is common when describing the interphase nucleus of a eukaryotic cell, when embryonic cells have homogeneous, diffuse chromatin, while in differentiated cells chromatin is dispersed throughout the entire volume and alternates with areas of highly condensed chromatin (Bostock and Sumner 1978; Koryakov and Zhimulev 2009).

Thus, before chromatin diminution, *C.kolensis* embryonic cells have a typical structure of interphase nuclei of embryonic cells, while after chromatin diminution, the structure of interphase nuclei irreversibly changes and more closely resembles the structure of interphase nuclei observed in multicellular eukaryotic cells after differentiation.

As is known maternal genes of the eggs determine the pattern of embryonic formation before fertilization and during initial cleavage divisions, after which the genes localized in the nuclei of embryonic cells play a role in the developmental process (Jaeger 2018). The similarity of the structure of *C.kolensis* somatic cells after diminution with differentiated cells of an adult organism may be due to changes in the structure of interphase nuclei in early embryogenesis in *C.kolensis* due to the transition from the regulation of maternal genes in the early stages of cleavage division to the regulation of nuclear genes of embryonic cells. These facts suggest that chromatin diminution as a stage of embryo development coincides with the stage of Maternal to Zygotic Transition, at which Zygotic Genome Activation occurs. With regard to the process of chromatin diminution itself, the question arises: what path does the initiation of chromatin diminution processes take? Is it through some factors present in the cytoplasm of an unfertilized egg, or do these factors appear in the presomatic cells of the embryo due to Zygotic Genome Activation. We hypothesized that if the chromatin diminution mechanism is triggered by nuclear genes, then suppression of the nuclear genome at the early stages of embryogenesis before the manifestation of the morphogenetic function of the nuclei should stop the chromatin diminution process; if the course of the diminution process is determined by cytoplasmic determinants, then inactivation of the nuclear genome will not affect the progress of the chromatin diminution process. This assumption was confirmed by data from an experiment on irradiation of *C.kolensis* embryos with high doses of radiation blocking the functioning of the nuclear genome of the embryos (Grishanin and Chinyakova 2021). The results of the experiment showed that mechanisms regulating the morphogenetic function of *C.kolensis* nuclei are triggered after the 4^th^ cleavage division, during which chromatin reduction occurs.

**Figure 1.**
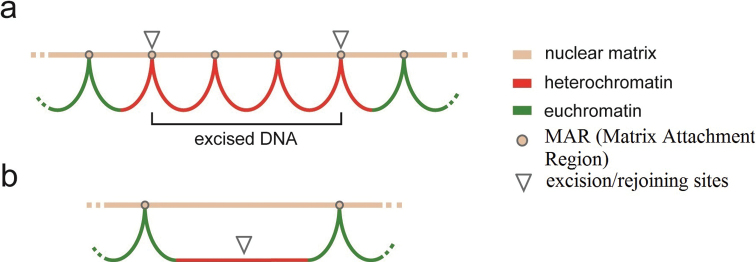
Hypothetical Matrix Attachment Region cutting scheme. Scheme of the *Cyclopskolensis* chromosome, in which, as a result of chromatin diminution, its part associated with the nuclear matrix (matrix attachment region, MAR) is cut out **a** chromosome before chromatin diminution **b** chromosome after chromatin diminution.

It has been established that during the course of chromatin diminution, a decrease in the size of the nuclei in the somatic line cells occurs (Beermann 1977; Grishanin et al. 1996; Gregory and Hebert 1999; Gregory 2001). According to the existing models of the organization of the eukaryotic interphase nucleus, all chromosomes occupy their strictly defined chromosomal territories, the functional activity of which is determined by the structure of these territories (Koryakov and Zhimulev 2009; Cremer and Cremer 2010). The ordered spatial arrangement of the intranuclear subcompartments of the interphase nuclei are generally evolutionarily conservative and genetically determined (Patroushev and Minkevich 2007). Interphase chromosomes are attached to the nuclear matrix, which is a network of protein fibrils to which chromatin strands are attached in areas called the Matrix Attachment Regions (MAR). For species with chromatin diminution, one might expect not only that the process of genome reduction is programmed, but that the structure of interphase nuclei, including the structure of the nuclear matrix, should also be rearranged as a result of chromatin reduction. In particular, a sharp decrease in genome size should coincide with a change in the number of permanent and functionally dependent sites for binding to the matrix of DNA molecules. A rearrangement of interphase nuclei structure after chromatin diminution can explain the results of certain experiments, which showed that the frequency of chromosome aberrations during post-diminution cleavage divisions in *C.kolensis* is 30–50 times less than during pre-diminution cleavage divisions (Grishanin and Akifyev 2005). A sharp decrease in the frequency of chromosome aberrations in embryos after chromatin diminution compared with embryos before chromatin diminution does not fit into the framework of the classical theory of chromosome aberrations induction (Savage 1989; Akifyev et al. 1990). Based on this theory, the frequency of chromosome aberrations in embryonic cells in *C.kolensis* before and after chromatin diminution should decrease in accordance with the reduction of the genome, in other words 15–16 times. However, the frequencies of chromosome aberrations in germ cells of *C.kolensis* before and after chromatin diminution differ by 50 times. The patterns discovered by Grishanin and Akifyev (2005) could be explained by the results obtained by Akifyev et al. (1995), according to which chromosome aberrations are formed in the minor part of the genome associated with Matrix Attachment Region, which is the most mutable part of the genome. The 94% of DNA removed from the somatic cell chromosomes in *C.kolensis* over the course of chromatin diminution is expected to include a significant number of Matrix Attachment Regions. If one assumes that most chromosome aberrations form in the part of the genome associated with nuclear matrix, then when this part of the genome is removed, the number of chromosome aberrations in cells should also decrease (Fig. 1). Hence, it can be assumed that chromatin diminution causes a 50-fold decrease in the number of points of contact between the nuclear DNA of *C.kolensis* presomatic cells and the nuclear matrix, as a result of which the frequency of chromosome aberrations is reduced by the same 50-fold.

Thus, chromatin diminution in copepods can be considered as a stage of irreversible differentiation of embryonic cells during ontogenesis. The reduction of 94% of the nuclear genome in *C.kolensis* makes it impossible to return the cells of the somatic line to the potencies of the germ line cells. ChroTeMo, a tool (Tkacz et al. 2016) for chromosome territory modelling, may be of great interest to those who study species with chromatin diminution.

## ﻿Chromatin diminution and C- value enigma

### ﻿History of the problem

The problem of non-coding DNA (*C*-value paradox, *C*-value enigma) was formulated in the middle of the 20^th^ century and relates to the fact that the most DNA of eukaryotic genomes is non-coding (Mirsky and Ris 1951; Dawkins 1976; Gregory 2001, 2005). While genomes of species belonging to the same genus, e.g., *Drosophilamelanogaster* Meigen, 1830 and *Drosophilavirilis* Sturtevant, 1916 (Moriyama et al. 1998), can differ in size by more than two-fold, there are no grounds or evidence that point to a significant difference in the number of genes between such species. Some amoebas have 200 times more nuclear DNA than humans, which does not indicate the presence of a larger number of genes in amoeba than in humans. Thus, the phenomenon of genome redundancy in eukaryotic organisms requires an explanation for the more than 200,000-fold differences in genome size that are not related to the complexity of the organism or the number of its genes. Many hypotheses have been proposed for the biological role of non-coding DNA. Some have not stood the test of time; others are still being discussed at the present time. So, the very first hypothesis of Callan (1967) was not supported, postulating that each gene consists of a series of tandem repeats, which are periodically checked for one copy to eliminate mutational divergence. This hypothesis did not stand the test of molecular genetics, since it was later found that eukaryotic genes are mainly represented by unique sequences. Some advocated for the idea of the regulatory function of non-coding DNA (Britten and Davidson 1971), believing that gene loci can be organized into operon-like structures (Georgiev 1970). Since the beginning of the 1970s, the opinion began to spread among biologists that the non-coding DNA has no function. The term “junk” in relation to non-coding DNA, was introduced by Ohno (1972). Ohno suggested that non-coding DNA does not affect the fitness of organisms, is a useless part of the genome, and is simply passively transferred by chromosomes to the next generation. The non-coding DNA came to be called “selfish” (Dawkins 1976). According to the authors of the “selfish” DNA hypothesis (Dawkins 1976; Doolittle and Sapienza 1980; Orgel and Crick 1980) an increase in the number of copies of sequences of the non-coding fraction of the genome with certain adaptive properties does not affect the phenotype and is not subject to selection. “Selfish” DNA can enhance their own transmission at the expense of other genes in the genome, even if this has no effect on organismal fitness. As a result, these authors believed that non-coding (“selfish”) DNA does not affect the adaptive properties of the whole organism. The prevailing point of view among molecular biologists is that non-coding DNA is selectively neutral (Charlesworth et al. 1994; Elder and Turner 1995; Kreitman 1996). Such DNA does not carry coding and regulatory functions, and although it is a certain metabolic burden for the organism, it is still not eliminated by selection and accumulates in the course of evolution as a result of mutational pressure. This concept is essentially similar to the “junk” DNA hypothesis. Petrov (2001) suggested that genome size fluctuations can occur under the influence of various factors: transposable genetic elements, degradation and excision of pseudogenes; the presence of “harmless” insertion sites, which equates events associated with changes in the structure of the genome occurring in these cases to neutral mutations. However, Petrov (2001) considers the change in the rate of appearance of small insertions and deletions (indel) to be the main factor in the variability of the size of the eukaryotic genome. If the frequency of spontaneous insertions and their size is greater than that of deletions, then, according to Petrov, this should create constant pressure in the direction of increasing the size of the genome. Ultimate control, according to Petrov, belongs to natural selection. With the weakening of selection, fluctuations in the size of the genome can be affected by other “factors”, for example, genetic drift, which can rebuild the genotypic structure of the population in a short time (the size of the genome in this case should be considered a phenotypic trait). This idea is consistent with concept of “skeletal” DNA known for more than 40 years (Cavalier-Smith 1978, 1985) and shared with certain reservations by some authors (Wyngaard and Gregory 2001; Kozlovski et al. 2003). According to the Cavalier-Smith concept, DNA not only encodes genetic information DNA but also has a structural function, and plays the role of a “nucleoskeleton” that determines the size of the nucleus, so the non-coding amount of DNA is determined by selection, since the larger the cell, the larger the nucleus should be. This correlation has been found for eukaryotes (Horner and Macgregor 1983; Olmo 1972; Gregory and Hebert 1999; Gregory et al. 2000) but not others (Pagel and Johnstone 1992). According to Gregory (2003), DNA plays not only a qualitative role in evolution, being a genetic material, but also a quantitative one, since changes in genome size should be considered as mutational events leading to phenotypic variations that can be influenced by natural selection. It should be noted that neither Gregory nor other authors reflecting on this topic believe that the nucleotypic hypothesis (Petrov 2001; Gregory 2003) is sufficiently substantiated and consistent.

Several authors have shown a correlation between genome size and various ecological or physiological parameters including the body’s resistance to cold and dryness in some plant species (Bennet 1987; Macgillivray and Grime 1995; Wakamiya et al. 1996), and the metabolic rate in certain species of mammals and birds (Vinogradov 1995). Vinogradov (1998) proposed the presence of buffer functions in non-coding DNA, providing passive energy-independent cell homeostasis, and would explain the dependence of the metabolic rate on the amount of non-coding DNA. The non-coding DNA is hypothesized to protects genes from the effects of physical and chemical mutagens (Hsu 1992). Of particular interest is a study on *Drosophila* Fallén, 1823, which showed a decrease in the viability of individuals as a result of the deletion of part of the satellite DNA (Wu et al. 1989). At various times, it was suggested that non-coding DNA is involved in the regulation of the functioning of unique genes, in particular, with the help of RNA interference (Fire et al. 1998). A hypothesis was proposed suggesting a protective function of non-coding DNA (Patroushev and Minkevich 2007).

The *C*-value paradox poses another question for biologists to answer: why organisms occupying a lower position on the phylogenetic tree, being ancestral forms or contemporaries of ancestral forms, have a significantly larger genome than more evolutionarily advanced or more specialized species. Mirsky and Ris (1951) drew attention to the fact that more specialized species have a smaller genome. Convincing evidence has been provided that animals and plants considered primitive or ancestral life forms, have more nuclear DNA than specialized species or species considered evolutionarily advanced (Ginatulin 1984; Hinegardner 1976). For example, psilophytic and fern-like plants contain up to 100 pg per haploid genome (1*C*), while the genomes of evolutionarily more advanced flowering plants contain less than 10 pg DNA per nucleus. While 90% of all modern fish species have a genome size in the range of 0.5–2 pg, the genome size of some species of Polypteridae have the range 3.69–7.25 pg, Salmonidae have the range 1.98–4.9 pg (www.genomesize.com). The genome size of the more primitive fish Chondrichthyes and Lepidosireniformes, which lived on the planet more than 400 million years ago, is much larger: in the former it is within the range of 1.58 – 14.8 pg, in the latter from 40 to 132.83 pg (www.genomesize.com). The genome size of caudate amphibians (Proteidae, Urodela) has a genome size from 25 to 120.6 pg per 1C (www.genomesize.com). Most bird species specialized for flight contain (0.91–1.93 pg DNA per 1C) 1.5–2 times less nuclear DNA than reptiles (1.26–5.44 pg per 1C), a genome size of Mammalia have in the range 1.63- 6.3 pg (www.genomesize.com). It cannot be expected that less specialized or ancestral species possess a large number of genes. The difference in the size of genomes depends on the amount of non-genic DNA. Therefore, non-coding DNA must perform a very specific function.

The study of the chromatin diminution process allows us to shed light on the fate of eliminated DNA (primarily constitutive heterochromatin), which was classified as non-coding or “junk”, and on the fate of some unique sequences that are also removed from the nuclear genome of somatic cells as a result of chromatin diminution. The idea of linking non-coding DNA to chromatin diminution belongs to Alexei Akifyev (Akifyev 1974; Akifyev et al. 2002; Akifyev and Grishanin 2005). He wrote: “Many years of dissatisfaction in understanding the biological role of non-coding DNA in eukaryotes, its actually dead-end state, from our point of view, is due to the fact that there was no directed search for that genetic process that would allow one to judge the actual functions of non-coding DNA and determine goals for further research.” According to Akifyev, the search for the biological role of non-coding DNA should be sought by studying the phenomenon of chromatin diminution.

A unique objective for solving the *C*-value enigma can be a representative of freshwater copepods, *C.kolensis*, in which, during the 4^th^ cleavage division, 94% of the DNA is excised from the chromosomes of somatic line cells, while germ-line cells retain their nuclear DNA unchanged throughout ontogeny. The diploid number of chromosomes remains unchanged (Grishanin et al. 1996; Drotos et al. 2022). In the somatic line, the remaining 6% of the genome is sufficient to perform all necessary functions of an adult organism. The eliminated 94% of DNA in *C.kolensis* can undoubtedly be considered as non-coding DNA for somatic cells, since the absence of this part of the genome in them does not interfere with the normal course of ontogenetic processes.

According to the selectively neutral hypotheses (Charlesworth et al. 1994; Elder and Turner 1995; Kreitman 1996), non-coding DNA has no coding or regulatory functions. It follows that the fraction of non-coding DNA, at least in fairly evolutionarily old species, should be dominated by sequences with a fairly high degree of divergence. In the eliminated DNA of *C.kolensis*, which we consider as non-coding for cells of the somatic line, there is a complex organization of various repeating sequences, due to the characteristic alternation of repeats and spacers, the complex structure of many repeats, the presence of slightly divergent, and often 100% identical to the consensus direct and inverted repeats present both in the same fragment and in different regions of the *C.kolensis* genome, many fragments (repeats) consist of submotifs, that is, they have a mosaic structure (Degtyarev et al. 2004). A comparative analysis of the consensus sequences of one of the eliminated DNA repeats *C.kolensis* showed that this repeat is present in the genome of both Moscow and Baikal populations of *C.kolensis* and is conserved (97–98% homology), is not eliminated completely in the course of chromatin diminution and is present in the genome of somatic cells of both populations (the degree of homology of the nucleotide sequence before and after diminution is 100% for the Moscow population and 99.1% for the Baikal population) (Grishanin et al. 2006c). It can be assumed that such strict conservation of non-coding sequences is determined by their role in the function of germline cells and does not allow us to consider the eliminated part of the genome as “junk” or “parasitic”.

The assumption that the role of non-coding DNA is in gene repression, which occurs during heterochromatinization of non-coding DNA, involving neighboring areas of euchromatin in this process (Zuckerkandl 1997), is unlikely from the perspective of data on copepods. Indeed, the elimination of 94% of DNA from cells of the somatic line of *C.kolensis* argues against this hypothesis, since morphogenesis begins after chromatin diminution is completed. Elimination of 94% of the genome of somatic cells of *C.kolensis* allows us to conclude that the eliminated DNA does not have significant coding and regulatory functions. Considering the fact that the full-length genome is preserved in germline cells, we hypothesize that some eliminated sequences, removed during the process of chromatin diminution from genome of somatic line cells, but retained in genome of germline cells, are necessary for the normal course of meiosis and maturation of germ cells.

## ﻿There is a need to dump non-coding DNA

Consider a model assuming the role of non-coding DNA in homologous recombination in *C.kolensis*. Suppose that the function of non-coding DNA is to increase the speed and frequency of recombination processes, the purpose of which is to increase the qualitative diversity of offspring that fall under the action of selection. At the same time, the more recombinant variants of the genome will be obtained, and the faster recombination events will take place, the more diverse offspring will be obtained. Given the interference rule, according to which two exchanges rarely occur in close proximity to each other, it can be assumed that the lower the density of genes in the genome, the higher the rate of recombination processes. It can be assumed that the role of non-coding DNA is to increase the distances between genes and their parts (exons and introns), as well as regulatory and structural elements (enhancers, silencers, insulators, MARs, etc.), in order to ensure the greatest freedom during recombination processes. The greater the distance between the coding regions of the genome, as well as coding and regulatory sequences, the greater the number of introns in the genes, and their magnitude, the less likely there will be violations of the structure of genes during recombination processes.

**Figure 2.**
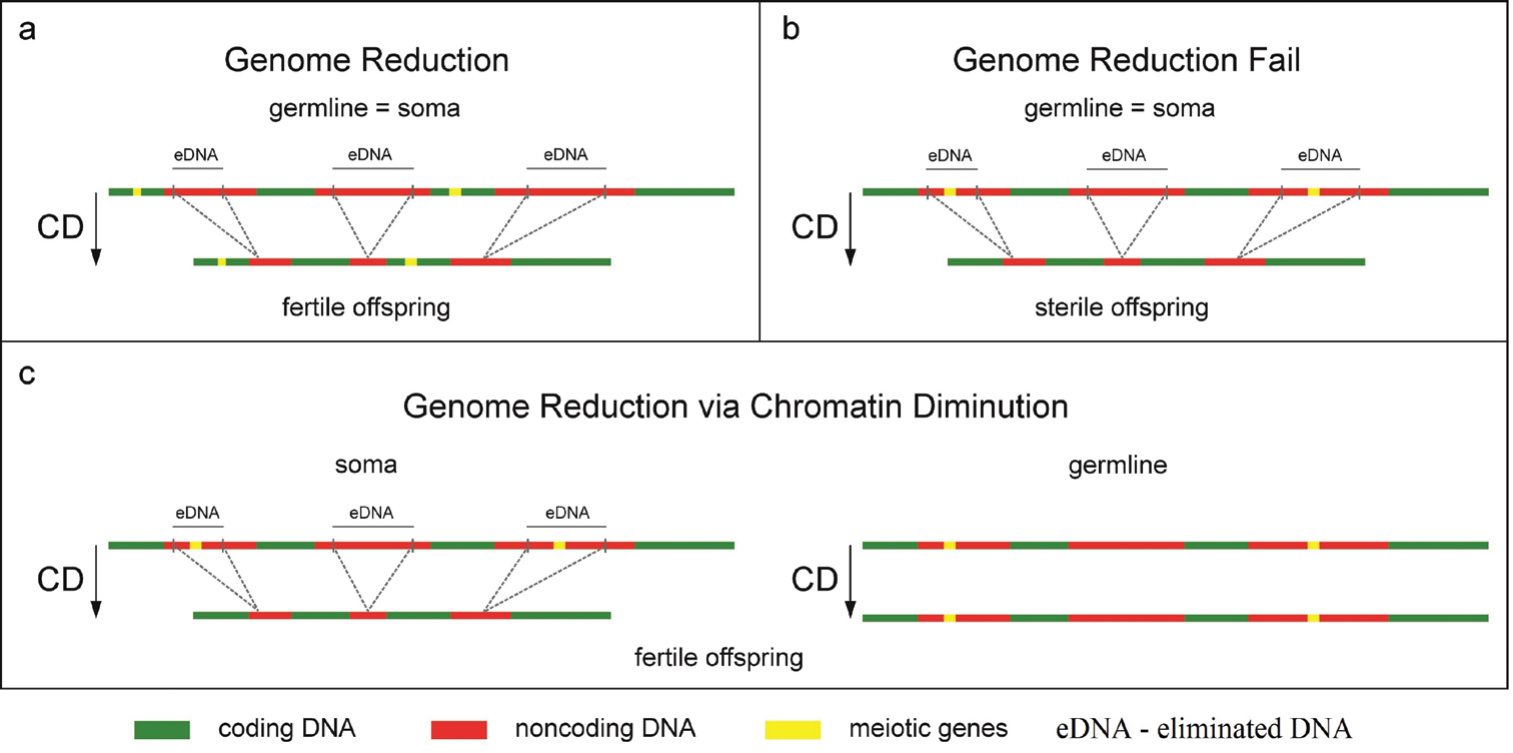
Hypothetical scheme of the origin of Chromatin Diminution (CD) **a** genome reduction during evolution occurs in germ line cells and somatic line cells; genes responsible for meiosis are located in the part of chromosomes that is not subject to genome reduction **b** genome reduction during evolution occurs in germ line cells and somatic line cells; during genome reduction the genes responsible for meiosis are located in the part of chromosomes that is subject to reduction; as a result, the offspring become sterile due to the absence of genes controlling meiosis **c** genome reduction occurs only in somatic line cells while preserving the original genome in germ line cells; during chromatin diminution the genes responsible for meiosis are located in parts of the chromosome that are subject to reduction during chromatin diminution; but they are retained in the chromosomes of germline cells. The offspring are viable. Chromatin diminution does not affect development processes.

The more often recombination events that take place, the more different gene variants will appear during the rearrangement of coding and regulatory sections of the genome, and the more variants of structural and regulatory proteins will appear in this individual. In addition, non-coding DNA, creating a spatial three-dimensional structure in the interphase nucleus, largely determines the genome’s likelihood to undergo ectopic recombination. Thus, a genome “diluted” with non-coding DNA makes it possible to quickly search for a wide variety of gene variants. The evaluation of these variants is carried out through the phenotype of an individual during the implementation of various genome variants in the interaction of the organism with the external environment. A successful variant of the genome should be stabilized; therefore, a decrease in the rate of recombination processes due to genome reduction can be considered as a mechanism for reducing genome variability. In other words, with the specialization and adaptation of the species to narrow ecological conditions, the need to find the optimal variant of the genome decreases. A large genome makes it difficult to fix the optimal variant of linear and spatial relationships of various parts of the genome, which allows the species to interact within this ecological niche in the most successful way. A non-coding genome during the fluctuation of the environment provokes further changes during recombination processes, and the loss of the optimal structure is found by it under the conditions of the ecological niche to which it has adapted. There is a need to dump non-coding DNA. This is achieved by genome reduction in somatic and germline cells. All the mechanisms necessary for such a process in cells exist: restriction by endonucleases and crosslinking of free ends by ligases. Genetic regulation of the main events of meiosis is well studied. If meiosis is disrupted, then sterility occurs in one or both sexes (Huang and Roig 2023). If, during recombination, important genes that should be involved during meiosis, but do not participate in the subsequent ontogenetic development of somatic cells, fall into the region of non-coding DNA intended for deletion, it becomes possible to preserve the original genome only in cells of the germline and reduce part of the genome in cells of the somatic line, which we observe in species with chromatin diminution (Fig. 2). Consequently, the origin of the chromatin diminution phenomenon can be considered as an incomplete process of genome reduction in both somatic and germline cells. In this case, chromatin diminution is an instrument of genome reduction in the course of evolution only in cells of the somatic line. Although the evolutionary advantages of a species with chromatin reduction are very conditional compared to a species without this phenomenon, nevertheless, this complex process of genome reorganization appeared during evolution. Despite the risk of losing important genetic information, species with chromatin diminution radically solve the problem of genome size reduction by removing, predominantly heterochromatin, from the genome of somatic line cells. Therefore, the chromatin diminution should not be considered as a rare phenomenon in the phylogeny of a small number of species, but as a universal mechanism of genome reduction, which may have been quite common among eukaryotes throughout their evolution. In addition, the removal of non-coding DNA during chromatin diminution can lead to a change in the sequence of exons and to a change in the level of gene expression. This point of view is consistent with the explanation of morphological evolution not due to the accumulation of point mutations, but due to the redistribution of genes, I.e. due to the rearrangement of DNA sequences and their exchange between members of the population (Doolittle and Sapienza 1980; Dover 1982).

## ﻿Chromatin diminution as a factor of genetic isolation

The appearance of chromatin diminution in the ontogenesis of a species of the genus *Cyclops* may also become a factor contributing to genetic isolation and further contribute to speciation. Due to the relatively short life cycle of freshwater copepods, genetic isolation can occur quite quickly (Dodson et al. 2003; Grishanin et al. 2005, 2006a). Investigating chromatin diminution in *C.kolensis*, we drew attention to the differences between the Russian and Germany populations of this species in a number of cytogenetic features and the chronology of diminution processes. According to cite author and year, chromatin diminution in individuals of the Moscow and Baikal populations of *C.kolensis* occurs during the 4^th^ embryonic division, and according to Ulrich Einsle, chromatin diminution in *C.kolensis* is observed during the 5^th^ embryonic division (Einsle 1993; Grishanin et al. 1996, 2006b). Granules of eliminated chromatin in the anaphase of diminution division of embryonic cells of individuals of the German *C.kolensis* population accumulate in the equator region, whereas specimens of the Russian *C.kolensis* population such granules accumulate mainly at the poles of the division spindle (Einsle 1993; Grishanin and Akifyev 2000). It is obvious that such signs as the presence or absence of chromatin diminution in ontogenesis, differences in the diploid number of chromosomes among the studied *Cyclops* species, differences in a number of characteristics of the chromatin diminution process (chronology of chromatin diminution, distribution of granules of eliminated chromatin in the anaphase of diminution division and other features of chromatin diminution) are inherited and rigidly determined in ontogenesis.

A large-scale rearrangement of the genome has occurred apparently in the species *Cyclopsinsignis* Claus,1857 as evidenced by the German population which has chromatin diminution (Einsle 1993) and the Russian population which lacks chromatin diminution (Grishanin et al. 2004); otherwise, has no visible morphological differences are evident. In this case, the mechanisms of speciation may be associated with the exclusion of those required stages of the diminution processes that must occur in presomatic cells in species that possess chromatin diminution, and the chromatin diminution process itself might thus be a driver of genetic isolation between populations that differ in how chromatin diminution is achieved, or between species, one of which has chromatin diminution and the other does not (Akifyev and Grishanin 1998, 2005; Grishanin 2014). The lack of morphological differences may be because cyclopoids, and especially the *Cyclops* genus, are characterized by morphological stasis. Analyzing the molecular structure of eliminated *C.kolensis* sequences (Akifyev et al. 2002) we assumed that eliminated DNA may play a role in the genetic isolation mechanism preventing the synapsis of homologous chromosomes in meiosis of interspecific *Cyclops* hybrids.

**Figure 3.**
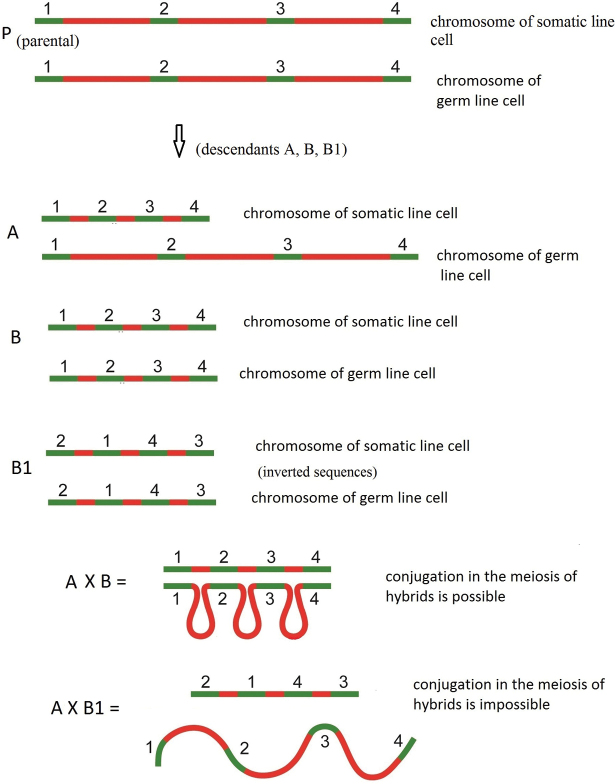
Hypothetical scheme in which reproductive isolation is determined by the appearance of chromatin diminution in one of the populations. Genome reduction of parental species (P) can take place in its descendants in two ways: population A, in which genome reduction occurred only in somatic line cells, and population B, in which genome reduction took place in somatic and germline cells. In population B due to inversion the linear order of the arrangement of functionally significant DNA sections has changed (population B1). In hybrids of individuals of populations A and B, partial conjugation will take place in meiosis. In hybrids of individuals of populations A and B1 conjugation in meiosis will be impossible and such hybrid individuals will be infertile.

Consider a hypothetical scheme of speciation. The parental species (presumably *Cyclops* sp.) has a genome containing a large amount of non-coding DNA (Fig. 3). The genomes of his somatic and germline cells are the same. Let us assume that during the evolution of a species a reduction of the genome is programmed. Genome reduction can take place in its descendants in two ways: population A, in which genome reduction occurred only in somatic line cells, and population B, in which genome reduction took place in somatic and germline cells (Fig. 3). At the same time, let’s assume that in population B due to inversion the linear order of the arrangement of functionally significant DNA sections, which the chromosomes of both the somatic and germ line possess, has changed and the exon sequences changed from 1-2-3-4 to 2-1-4-3. Individuals with inverted chromosomes will be denoted as population B1 (Fig. 3). Individuals of population A, in which genome reduction took place only in cells of the somatic line, retained the original linear order of the arrangement of functionally significant sections of the genome. When crossing individuals of population A with individuals of population B or B1, they will give different pictures of the chromosomes conjugation in meiosis. In hybrids of individuals of populations A and B, partial conjugation will take place in meiosis (Fig. 3). In hybrids of individuals of populations A and B1 conjugation in meiosis will be impossible, as a result of which meiosis will be disrupted, and such hybrid individuals will be infertile (Fig. 3). In other words, a genetic barrier will arise between hybrid individuals of populations A and B1. Therefore, we can assume that chromatin diminution and genome reorganization may lead to genetic isolation of individuals (populations) of cyclops species.

The phenomenon of gonomery in fresh-water Copepoda species can be considered as an example of an intermediate stage of genome evolution in species with the phenomenon of chromatin diminution. Sigrid Beermann (1977) found polymorphism in the amount of heterochromatin in females of *C.strenuusstrenuus* and *Cyclopsfurcifer* Claus, 1857. Dimorphism in the content of heterochromatin in *C.s.strenuus* also causes a difference between the sexes. If the females are *C.s.strenuus*, as a rule, are heterozygous for the “enrichment” of chromosomes with heterochromatin, then males are always homozygous for this trait and contain only large chromosomes with interstitial heterochromatin. In heterozygous females, chromosomes enriched with “heterochromatin” from a set of large chromosomes and a set of small chromosomes, which consist primarily of euchromatin diverge in separate groups during anaphase of cleavage divisions before chromatin diminution (Beermann 1977). About half of the eggs have one set of short chromosomes and one set of long ones, while the other half of the eggs contain only long chromosomes. The difference in length is approximately equally distributed between all chromosomes. A smaller amount of eliminated chromatin is formed in heterozygous embryos, a larger amount of eliminated chromatin in homozygous ones. Removal of a part of chromatin from the chromosomes of *C.s.strenuus* as a result of chromatin diminution leads to a decrease in the size of chromosomes. Chromosomes enriched with heterochromatin from a set of large chromosomes change more strongly than chromosomes from a set of small chromosomes, which consist primarily of euchromatin. In species of *C.s.strenuus* and *C.furcifer* chromatin diminution completely eliminates the significant difference in size between homologous chromosomes. In other words, chromosomal polymorphism is limited only to germline cells. Regardless of the distribution of eliminated chromatin in all three species *C.s.strenuus* and *C.furcifer* after chromatin diminution there are always 22 pairs of identical chromosomes in diploid somatic cells. Gonomery and chromatin diminution was also found in *Mesocyclopslongisetus* Forbes, 1891 (Copepoda) (Rasch and Wyngaard 2008). Chromosomes from the set of small chromosomes in females of *C.s.strenuus*, in which there is no eliminated chromatin, can be considered as a genome in which a reduction of heterochromatin (part of non-coding DNA) has occurred. Thus, we can consider species with gonomery as an example of genome evolution, during which genome reduction is observed not only in somatic line cells, but also in germ line cells.

## ﻿Conclusions

Reduction of 94% of DNA in the somatic cell line as a result of chromatin diminution in *C.kolensis*, allowed us to consider the eliminated DNA as non-coding for cells of the somatic line, since the absence of this part of the genome in them does not interfere with the normal course of ontogenesis. At the same time, it suggests that the eliminated DNA does not carry any significant coding and regulatory functions in the somatic line.

Studies of chromatin diminution in *C.kolensis* have shown that as a result of chromatin diminution, a change in the structure of interphase nuclei occurs, which is characteristic of the interphase nucleus of a differentiated eukaryotic cell. The results obtained led to the conclusion that the process of chromatin diminution is an alternative form of regulation of cell differentiation into the somatic and germ lines.

Studies of different species of *Cyclops* have shown that the reason for the appearance of chromatin diminution in ontogenesis is not related to the need to remove non-coding DNA from the genome of somatic cells, as can be seen when comparing *C.insignis* from Moscow, Russia, which does not have chromatin diminution, and *C.insignis* from Germany, which has chromatin diminution (Grishanin et al. 2004).

The genome reduction is a tool aimed at reducing the speed of the evolutionary process of a species by reducing the frequency of recombination events, which leads to a decrease in the diversity of genotype variants in offspring when the necessary level of adaptability to environmental requirements is achieved. The chromatin diminution can be considered as one of the options for this process, when genome reduction in germ line cells is impossible due to localization of sequences there that are presumably important for the processes of meiosis and early stages of embryogenesis, but not necessary for subsequent development.
